# Remarks on Lupulin as an Anaphrodisiac

**Published:** 1849-05

**Authors:** Wm. Byrd Page

**Affiliations:** Consulting Surgeon to the Philadelphia Hospital, Blockley


*Remarks on Lupulin as an Anaphrodisiac.* By Wm. Byrd Page, M. D., Consulting Surgeon to the Philadelphia Hospital, Blockley.

In offering to the medical profession the application of a means of relief for any affection, the practitioner does nothing more than a self-imposed duty requires.

Actuated only by such a motive, I propose the administration of Lupulin as an anaphrodisiac, a use to which, I believe, the article has never before been applied as a therapeutic agent.

The hop has long held a prominent position in the materia medica as a tonic, and as a narcotic and calmant, in many disordered conditions of the nervous system. Its different preparations have been administered internally, and applied externally, in affections calling for the exhibition of medicines for the production of sleep, the relief of pain, and for quieting unusual nervous excitement, when the more powerful and usually more certain medicines of the same class, have been deemed inadvisable from some peculiar circumstances.

The principal virtues of the hop are believed to reside in Lupulin, which is described as occurring as a secretion in the form of granules, on the under surface of the scales or strobiles of the Humulus lupulus.

Lupulin was first described, and its properties made known, by Dr. A. W. Ives, of New York, though some notice had been previously taken of it. According to the U. S. Dispensatory, " it is obtained, separate, by rubbing or threshing and sifting the strobiles, of which it constitutes from one-tenth to one-sixth by weight. It is in the state of a yellowish powder, mixed with minute particles of the scales, from which it cannot be entirely freed when procured by a mechanical process. It has the peculiar flavour of hops, and appeared to MM. Lebaillif and Raspail, when examined by the microscope, to consist of globules filled with a yellow matter, resembling in this respect the pollen of vegetables. It is inflammable, and when moderately heated becomes somewhat adhesive."

It is kept by most apothecaries, and can be procured in a few moments by the above simple process.

More than two years since I introduced Lupulin to a limited extent into the Philadelphia Hospital, (Blockley,) as a remedy to prevent nocturnal erections in different forms of acute venereal disease, and have subsequently used it sufficiently often in my practice, to justify its presentation to the medical profession as a very good article for the purpose, one of great efficacy, and entirely free from many of the objections to the preparations of camphor, opium, dulcamara, stramonium, &c., which have hitherto been principally relied on.

One of the most painful and troublesome attendants upon gonorrhœa, is chordee, brought on by nocturnal erections, the occurrence of which has been completely prevented by the administration of Lupulin at bed time.

In acute gonorrhœa, it not only prevents erections and consequently chordee at night, but it also seems to exercise a very soothing effect on the inflamed urethra, and to facilitate the operation of medicines for the cure of the disease.

Relief from the troublesome pain in the perineum in chronic gonorrhœa, and during the treatment of stricture with the bougie, has been obtained by the administration of Lupulin alone.

In the treatment of chancres on the penis, the process of healing is often interfered with, and the efforts of nature and the surgeon placed somewhat at defiance by the occurrence of erections, when the patient is warm in bed, which distend the parts and lacerate the edges of a weak or imperfectly formed cicatrix. In this disease the Lupulin has been used with the desired effect.

I have also used it after the operation for phymosis, with the effect of preventing the occurrence of erections during the process of the cicatrization of the incision. Its use may doubtless be adopted with the same intention after any other operation on the penis.

The Lupulin has been administered for nocturnal seminal emissions, and although it does not claim a curative power in this distressing affection, it will prevent their occurrence so long as the patient is freely under its influence, and will give the practitioner an opportunity to prosecute any treatment which he may adopt, with an increased prospect of success, from the interruption to the habit of the disease, and from the prevention of erections when topical applications are made to the urethra. I cauterized the prostatic and membranous portions of the urethra, with Lallemand's instrument, for a gentleman labouring under this disease, and gave Lupulin to prevent erections, which often harass the patient after this simple operation, with complete success.

My own experience in the use of the remedy has been corroborated by that of other practitioners, who have given it at my suggestion. Dr. F. G. Smith administered it to a patient suffering from spermatorrhœa, and prevented the recurrence of the emissions so long as the effect of the remedy was kept up. He has also given it to a gentleman under his care with chancre. Dr. Edward Hartshorne reports to me a case which establishes its efficacy beyond a doubt, in suppressing the venereal appetite. A healthy negro man confined in the Eastern Penitentiary, practised onanism to such an extent, as to bring on an attack of insanity. The mania was relieved by active treatment, and the usual means were applied for the suppression of what seemed his ruling passion, without effect. He became conscious of his unfortunate condition, and of its cause, and confessed that he passed his time when not watched, in this self-debasing, and health-destroying amusement. He entreated the Dr. to give him something " to take his courage down." Lupulin was administered to him in two grain doses, several times in the twenty-four hours, and he now states that " it is all gone," and that he is no longer troubled with his hitherto unconquerable desire.

The dose of Lupulin is from 5 to 10 grains, to be repeated as occasion requires. The latter dose rarely requires a repetition during the night. It may be given in powder or in pill. It produces no headache, does not constipate, or give rise to nervousness or any other unpleasant consequence.

